# Use of the STONE Score for Predicting the Outcomes of Lithotripsy: A Narrative Review

**DOI:** 10.7759/cureus.90570

**Published:** 2025-08-20

**Authors:** Ifeanyichukwu E Ihedoro, Paul E Ngwu, Chiamaka H Obodozie, Chibueze P Ohiarah, Enyinnanya V Onyemachi

**Affiliations:** 1 Urology, Eleos Specialist Hospital, Umuahia, NGA; 2 Urology, North Cumbria Integrated Care NHS Foundation Trust, Carlisle, GBR; 3 Urology, Federal Medical Centre, Umuahia, Umuahia, NGA; 4 Radiology, Olabisi Onabanjo University Teaching Hospital, Sagamu, NGA; 5 Surgery, Federal Medical Centre, Umuahia, Umuahia, NGA

**Keywords:** eswl (extracorporeal shockwave lithotripsy), percutaneous nephrolithotomy (pcnl), stone nephrolithometry score, stone score, ureteroscopic lithotripsy

## Abstract

Urinary stone disease is a common urological condition that affects individuals across all age groups worldwide. It is traditionally more common in developed countries, but the incidence is rising in the tropics. This increase is possibly linked to climate change, increased ambient temperatures, and the adoption of westernized diets. Its occurrence is approximately twice as high in males as in females. Several systems have been formulated to help predict complications and other outcomes of lithotripsy. Examples include the STONE (Size, Topography, Obstruction, Number, Evaluation) scoring system, Clinical Research of the Endourological Society nomogram, Guy’s scoring system, and Seoul National University Renal Stone Complexity. The STONE scoring system is a simple tool derived from CT findings of patients with upper urinary tract stones. It was initially designed to predict the stone-free rate in patients undergoing percutaneous nephrolithotomy. However, it was later modified for use in ureteroscopy. Multiple studies have been published that show that the score has a reasonable predictive accuracy in determining the outcomes of lithotripsy. It is comparable to the other nephrolithometric scoring systems and can serve as a useful tool in perioperative patient management. This review aims to examine the STONE scoring system, its correlation with clinical outcomes, and comparison with alternative scoring systems, ultimately assessing its importance in the management of patients with upper urinary tract stones.

## Introduction and background

Urinary stone disease is a common urological condition that affects individuals across all age groups worldwide. Historically, it was more common in developed countries, but the incidence is rising in the tropics. This increase is possibly linked to climate change, increased ambient temperatures, and the adoption of westernized diets. Its occurrence is approximately twice as high in males as in females [1,2]. It can occur in any part of the urinary tract. The urinary tract can be divided into the upper (kidneys and ureters) and the lower (bladder and urethra) urinary tract [3]. Several treatment modalities exist for managing upper urinary tract stones, including percutaneous nephrolithototomy (PCNL), ureteroscopy (URS), and shockwave lithotripsy (SWL), among others. [4] Several systems have been formulated to help predict complications and other outcomes of lithotripsy, including the STONE (Size, Topography, Obstruction, Number, Evaluation) scoring system, the Clinical Research of the Endourological Society (CROES) nomogram, Guy’s scoring system (GSS), and Seoul National University Renal Stone Complexity (S-ReSC) [5-7]. This review aims to detail the components of the STONE score, examine its correlation with clinical outcomes, and compare it to alternative scoring systems. Ultimately, we aim to discuss its significance in the management of patients with upper urinary tract stone disease.

## Review

Methodology

This narrative review gathered primary resources through PubMed, MEDLINE via Ovid, ScienceDirect, and Google Scholar searches. Publications from peer-reviewed journals from 1993 to 2025 were reviewed. No systematic review methodology (such as the Preferred Reporting Items for Systematic Reviews and Meta-Analyses) was applied. Only publications written in the English language were included. Search terms included lithotripsy, urolithiasis, STONE score, stone-free rate, and predictive outcomes.

The STONE score: definition and components

The STONE score was originally developed by Okhunov et al. to provide clinicians with a simple tool for predicting outcomes of PCNL using features noted on CT obtained before surgery [8]. It was formulated from variables already known to influence the outcomes of lithotripsy. The variables of the score included the stone size (mm), tract length (mm), presence of obstruction, number of calyces involved, and the Hounsfield unit of the stone (HU). The score ranges from 5 to 13, with a score of 5 indicating the least complex stone and a score of 13 the most complex stone [8]. Molina et al. modified the STONE score to assist in the management of patients undergoing ureteroscopy with laser lithotripsy [9]. The STONE score, as described by Molina et al., comprises five parameters, i.e., size of the stone, topography or location of the stone, presence of obstruction, number of stones, and the evaluation of the Hounsfield unit. Each parameter is given a number of 1 to 3, resulting in a total value of 5 to 15 [9]. Patients may often present with multiple stones in the kidney or ureter. In such cases, the calculi with the highest score in each parameter are used in calculating the score. Table 1 and Table 2 illustrate the components of the STONE system and how each parameter is scored [8,9].

**Table 1:** STONE (Size, Topography, Obstruction, Number, Evaluation) score as described by Okhunov et al. The table has been modified from Okhunov et al. [8].

Feature	1 point	2 points	3 points	4 points
Stone size (mm^2^)	0–399	400–799	800–1.599	≥1,600
Tract length/skin to stone distance (mm)	≤100	>100	-	-
Obstruction/hydronephrosis	None	Severe	-	-
Number of calyces involved	1–2	3	Staghorn calculus	-
Evaluation of Hounsfield units	≤950	>950	-	-

**Table 2:** STONE (Size, Topography, Obstruction, Number, Evaluation) score as described by Molina et al. The table has been modified from Molina et al. [9].

Variable	1 point	2 points	3 points
Stone size (mm)	<5	5–10	>10
Topography/location of stone	Distal to mid-ureter	Proximal ureter through the mid and upper pole	Lower pole
Obstruction/hydronephrosis	Preoperative stent or no hydronephrosis	Grade 1–2	Grade 3–4
Number of stones	1	2	≥3
Evaluation of Hounsfield units	<750	750–1,000	>1,000

Several studies have shown that stone size and volume have a strong ability to predict stone clearance following lithotripsy [10]. On the other hand, Yang et al. [11] showed that stone size and location were important predictive factors for stone clearance. The larger the size of the stones, the lower the rate of stone clearance following lithotripsy. Molina et al. described stone size as the widest diameter in any plane on non-contrast abdominal CT imaging, while Okhunov et al. defined this variable as a multiplication of the stone width and length in square millimeters [8,9]. Studies have shown that stone size has a correlation with the outcome of surgery. In a study conducted on 155 patients who underwent extracorporeal shockwave lithotripsy (ESWL), patients with a stone size of 5-10 mm had a success rate of 68.4% while those with a size >10 mm had a success rate of 32% [1]. A study by Yılmaz et al. reported that stone size was an important predictor of the stone-free rate [12].

The topography describes the location of the stone within the ureter or kidney. A study by Daly et al. showed that clearance rates following ureteroscopy were higher in patients with lower ureteric stones (72%) compared to mid and upper ureteric stones (61% and 38%, respectively) [13]. Lithotripsy for stones in the lower pole of the kidney is more challenging compared to stones in the upper and middle poles due to the acute infundibular angle [9]. In a study conducted on 86 patients who had renal stones and underwent URS with laser lithotripsy, upper pole kidney stones had 100% clearance, middle pole stones had 95.8% clearance, while lower pole stones had 90.9% clearance [14].

Studies have described the negative impact of hydronephrosis on the outcome of lithotripsy, with higher grades of hydronephrosis increasing the risk of poor stone clearance and postoperative complications [15,16]. The grading system of hydronephrosis used is a modification of the Society for Fetal Urology Hydronephrosis Grading System [17]. In a study conducted on 259 patients who underwent supine PCNL for renal stones, the stone-free rate was noted to reduce with worsening hydronephrosis. The stone-free rate was 86.6% in patients without hydronephrosis, 82.5% in patients with mild hydronephrosis, 76.0% in patients with moderate hydronephrosis, and 61.5% in patients with severe hydronephrosis [15].

The number of stones is an important determinant of stone clearance after lithotripsy. Normograms predicting stone-free rate three months after lithotripsy showed that patients with multiple calyceal stones of above 2 cm in size had a significantly lower chance of stone clearance (10.5%), showing that stone number and stone size are crucial factors in predicting the outcome of lithotripsy [18].

The Houndsfield unit is known to have an impact on the rate of stone clearance following lithotripsy. In a study by Al-Zubi et al., 155 patients underwent ESWL for renal and ureteric stones. The results showed that stone clearance increased with reducing stone density. Patients with a stone density of less than 500 HU had a success rate of over 90% following ESWL [1]. High HU is, therefore, known to lower the success rate of lithotripsy [19].

The tract length (skin to stone distance) is also an important predicting factor of stone-free rate and complications after PCNL. A long tract length can be associated with difficulty in assessing the kidney and dilation of the tract. It may also cause difficulties in securing the sheath and movement of the nephroscope during the procedure. This can lead to a prolonged surgery, low stone-free rate, and increased complications [20,21].

The number of calyces involved is related to stone burden and can affect the rate of stone-free rate and surgical outcome. It is associated with longer surgical times, stone-free rate, and estimated blood loss [8,22].

A study done by Kim et al. showed that the location, size, number, Hounsfield unit, and severity of hydronephrosis were important predictors of the outcome of SWL [23]. With several studies showing that different components of the STONE score have a significant correlation with outcomes of lithotripsy [24], it is thought that a combination of these parameters would increase the predictive accuracy of the system.

Predictive value of the STONE score in urolithotripsy

Ahmed et al. conducted a retrospective, single-center study among 266 patients with ureteral stones who underwent lithotripsy for ureteric calculi. They reported that the score had a high predictive accuracy for stone-free rate with an area under the curve (AUC) of 0.785 [25]. Sirirak et al. [5] evaluated 155 patients who had undergone URS lithotripsy for ureteric calculi and noted similar results with an AUC of 0.815 for predicting stone-free status post-URS. Richard et al. conducted a similar study on 800 patients who had undergone URS lithotripsy for renal and ureteric calculi. They reported that the STONE score was predictive of stone-free rate with an AUC of 0.617 [26]. Molina et al [9], in their study among 200 patients who had undergone URS lithotripsy for renal and ureteric calculi, reported an AUC of 0.764 for predicting stone-free status post-URS, while Shrestha et al. [27] reported an AUC of 0.693 for predicting stone-free status post-URS. Jasser et al. [28] in Tunisia conducted a study among 92 patients who had undergone URS lithotripsy for ureteric calculi. They reported an AUC of 0.73 for predicting stone-free status post-URS. A similar finding was noted by Selmi et al., who reported an AUC of 0.725 for predicting stone-free status post-URS [29]. These findings consistently show that the STONE score is predictive of the stone-free rate in patients undergoing URS and lithotripsy.

The STONE score has been known to show a good correlation with the stone-free rate following PCNL. In a novel study by Okhunov et al., results showed that the STONE score correlated with the stone-free rate and was an important determinant of postoperative complications, postoperative transfusion rate, and hospital stay [8]. Biswas et al. performed a prospective, single-center study among 252 patients who underwent PCNL. They reported that the STONE score was predictive of the stone-free rate with an AUC of 0.852 for predicting stone-free status [30]. Yarimoglu et al. in Turkey noted similar findings. They reported a significant correlation between the STONE score and stone-free rate among 567 patients who underwent PCNL for renal stones [31]. Similarly, Illahi Bux et al. [32] reported an AUC of 0.680 for predicting stone-free status post-PCNL among 147 participants who underwent PCNL for renal stones, while Noureldin et al. [33] reported an AUC of 0.63. Several studies have validated the STONE model [34-36] and have shown that it is an effective tool in predicting postoperative outcomes following PCNL.

Several studies have reported varying results when correlating the STONE score with complications of lithotripsy. Sirirak et al. in their study reported a significant correlation with operative time, length of hospital stay, and presence of complications in patients who underwent URS lithotripsy [5]. Biswas et al. noted that the STONE score significantly correlated with blood loss, while Yarimoglu et al. reported that the STONE score significantly correlated with operation time and complication rates among patients who underwent PCNL [30,31]. On the other hand, Illahi Bux et al. [32] and Noureldin et al. [33] noted that there was no significant correlation between the STONE score and the modified Clavien grading system.

In an earlier study conducted by Tailly and Razvi in 2016, which assessed 586 patients, the STONE score could predict a stone-free rate with an AUC of 0.671. Moreover, it was found to be an independent predictor of longer operation time. The findings from this study placed the STONE score on par with other nephrolithometric tools, such as the Guy’s and CROES scores, in predicting stone-free rates while also suggesting its efficacy in estimating procedural difficulty with prolonged operation time [37].

Senocak et al. conducted a study to evaluate the predictive value of GSS in determining residual stone rates and complications for pediatric patients undergoing PCNL [38]. The study included a cohort of 114 children treated at a single tertiary institution. The study noted a significant association between higher GSS and lower stone-free rates, as well as increased postoperative complications. Specifically, the study found that although GSS had a significant predictive ability for residual stone rates after pediatric PCNL, the results did not show that the complexity of GSS was directly proportional to complications, with factors such as the number of calyceal involvements and surgeon experience proving additional points to consider in predicting outcomes. The study, however, underscored the utility of nephrolithometric scoring such as the GSS in pediatric populations.

Okhunov et al. in 2013 published a study on the use of the STONE nephrolithometry. In this study, a cohort of 117 patients was included, which showed the efficacy of the STONE score to predict treatment and risk of perioperative complications after PCNL [8].

A recent study by Saeed et al. (2024) evaluated the predictive value of the STONE nephrolithometry score in determining the need for blood transfusion among patients undergoing PCNL [39]. Conducted as a prospective observational study involving 89 patients, the researchers reported a mean STONE score of 7.87 ± 1.54 across the cohort. Notably, patients who required perioperative blood transfusion exhibited significantly higher STONE scores compared to those who did not, suggesting a strong correlation between nephrolithometric complexity and intraoperative hemorrhagic risk. The study concluded that the STONE score could serve not only as a predictor of stone-free rates but also as a useful preoperative tool in identifying patients at elevated risk of bleeding, thereby informing perioperative planning and resource allocation.

A study conducted by Handa et al., including a total of 100 patients who underwent PCNL, aimed to establish the relationship between the Guy’s and STONE scores in determining their predictability for postoperative stone-free rates and complications. The study showed that both scoring systems demonstrated positive correlation with stone burden, operation time, and blood loss. The overall stone-free rate was 72% for both GSS and STONE and was significantly associated with the success of the procedure [40].

A small comparative study assessed the application time of scoring systems. The STONE score required approximately five minutes to compute, compared to under one minute for the GSS. Despite the longer calculation time, predictive accuracies for stone-free rates were similar. However, the added detail captured by the STONE parameters (e.g., HU, calyceal involvement) might justify its use in complex cases requiring nuanced risk stratification [41].

Malik et al. conducted a prospective validation study in a Pakistani tertiary care center to evaluate the diagnostic accuracy of the STONE clinical prediction score in identifying ureteral stones in patients presenting with flank pain [42]. The study enrolled 134 patients and reported an AUC of 0.752, indicating good predictive performance. Higher STONE scores were significantly associated with the presence of ureteral stones confirmed on imaging. The authors concluded that the STONE score is a practical and non-invasive triage tool that can assist emergency physicians in risk-stratifying patients into low, moderate, and high risk, as well as in determining the need for further investigation, especially in resource-limited settings.

A large multicenter study involving 845 patients across nine emergency departments evaluated the STONE score for non-contrast CT-based triage of ureteral stones. High STONE scores were strongly predictive of the presence of ureteral calculi (AUC ~0.78) and were more accurate than physician gestalt (AUC ~0.68) [7]. Additionally, Moore et al. conducted both retrospective and prospective observational cohort studies. The study included 491 patients and concluded that the STONE score reliably predicts the presence of uncomplicated ureteral stone and a lower likelihood of acutely important alternative findings. It also suggested that the incorporation of the STONE score in future investigations may help limit exposure to radiation and overutilization of imaging [43].

In a study by Kim et al., the external validation of the original STONE score and the derivation of a modified version were undertaken to improve the prediction of ureteral stone presence in emergency settings [44]. This retrospective analysis included 700 patients who underwent non-contrast CT for suspected ureteral colic, and 555 had a ureteric stone. The modified STONE score incorporated additional clinical variables and yielded an improved AUC compared to the original version. The study demonstrated that both versions of the STONE score are useful diagnostic aids, with the modified version offering slightly enhanced accuracy. These findings support the role of structured clinical prediction tools in streamlining emergency department evaluations for renal colic.

Clinical utility of the STONE model

The STONE score can serve as an important tool in preoperative counselling of patients, providing an objective tool for assessing the likelihood of complete stone clearance and the risk of postoperative complications [34,45]. As Sirirak et al. noted, scoring systems in lithotripsy are vital in surgical practice, as they “help with risk assessment for patient counselling, surgery preparation, prediction of the postoperative phase, and standardized reporting of the outcome measures” [5]. The STONE score provides clinicians with an objective tool during patient counselling. Quality counselling helps patients have realistic expectations regarding the outcomes of their surgery, including, rate of stone clearance, repeat procedures, and postoperative complications.

Beyond counseling, the STONE score plays a valuable role in surgical logistics and resource allocation. It allows clinicians to stratify patients based on complexity and prioritize cases accordingly. High-complexity cases, as indicated by elevated STONE scores, may necessitate more experienced surgeons, longer operative time, specialized equipment, or availability of blood products. These patients might also benefit from being scheduled in high-volume centers with advanced endourological expertise.

In patients with a more complex STONE score, decisions can be made to refer to a more specialized hospital, thereby providing the patient with a chance of a more optimal care. It is, therefore, useful in planning intra- and postoperative care for patients undergoing lithotripsy. Validation of the STONE score can also be used in comparing outcomes of laser lithotripsy among different surgeons because it offers an objective and uniform way of reporting outcome measures. This makes the description of patient characteristics better and improves the standard of data reporting among urologists [6,46]. Finally, as the score relies on CT images of patients for its computation, the STONE scoring system is simple to use, relatively cheap, and can be utilized in low-resource environments [32]. This score can, therefore, be applied to clinical practice, serving as an important tool in the care of patients with upper urinary tract stones.

An essential aspect of modern surgical practice is the standardization of outcome measurement and benchmarking of performance. The STONE score offers a reproducible and quantifiable method to characterize the preoperative complexity of renal and ureteric stones. It allows outcomes such as stone-free rates, operative time, complications, and hospital stay to be interpreted in context. Without adjustment for case complexity, outcome comparisons between different surgeons, institutions, or countries may be misleading.

This objectivity in preoperative assessment enhances academic research and facilitates multicenter studies by establishing a common language for reporting and analyzing results. Surgeons and departments can use STONE scores to audit performance, compare results over time, and identify factors contributing to variation in outcomes. It also contributes to surgical training by allowing residents and fellows to understand how anatomical and radiological features influence procedural difficulty.

Moreover, its reliance on universally available imaging data means it does not require additional tests or costly software platforms. As a result, clinicians in settings with limited subspecialty access can use it to stratify cases, refer appropriately, and optimize outcomes. It bridges the gap between advanced urological care and practical decision-making in developing regions.

Multiple studies have demonstrated the reliability of the STONE score in predicting surgical outcomes. For instance, Molina et al. validated the score for URS, reporting a significant correlation with stone-free rates [9]. Similarly, Okhunov et al. showed strong associations between the score and outcomes such as transfusion rate, complications, and hospital stay following PCNL [8]. The predictive accuracy for stone-free rates has been consistently confirmed by various authors across different settings and populations.

While there is robust evidence linking the STONE score with stone clearance rates, the correlation with complications is more variable. Some studies, such as that by Biswas et al., noted a significant relationship with blood loss and operative time, while others found no strong association with the Clavien-Dindo complication grading system [30,32]. This variability highlights the need for further studies to refine the score’s role in predicting complications and to potentially incorporate adjunctive factors such as body mass index, renal anatomy, or prior surgical history.

The STONE score also serves as an effective teaching tool for medical students and surgical trainees. It encourages systematic interpretation of imaging, consideration of anatomical complexities, and prediction of surgical challenges. By using the score in educational discussions, trainees learn to correlate imaging findings with clinical implications and procedural planning.

In research, the score offers a valuable endpoint for prospective trials or retrospective analyses. It allows investigators to stratify cohorts, adjust for confounding variables, and standardize inclusion criteria. This helps ensure methodological rigor and improves the comparability of findings across studies.

Despite its advantages, clinicians must be aware of the limitations in its clinical application. Variability in imaging protocols, subjective assessment of obstruction, and differences in how Hounsfield units are measured can introduce interobserver variability. Additionally, the score does not account for certain anatomical anomalies (e.g., horseshoe kidney, ureteropelvic junction obstruction) that may independently influence surgical outcomes.

It is also important to note that the STONE score does not predict patient-specific factors such as pain threshold, adherence to follow-up, or socioeconomic considerations, all of which can affect treatment success and patient satisfaction. Nevertheless, when used alongside clinical judgment, it remains an indispensable adjunct in the urological toolkit.

The STONE score provides a practical, validated, and efficient method for assessing the complexity of urinary stones and predicting surgical outcomes. Its utility spans the entire continuum of care, from diagnosis and counselling to operative planning, outcome reporting, and academic benchmarking. With increasing adoption in clinical and academic settings worldwide, the STONE score is well-positioned to remain a central tool in the management of nephrolithiasis, particularly as precision medicine and data-driven healthcare continue to evolve.

Comparison of the STONE score with other stone scoring systems

Several studies have compared the STONE score with similar scoring systems with the aim of comparing their effectiveness in predicting outcomes of lithotripsy. Other common models in practice include the CROES nomogram, GSS, and S-ReSC [5-7]. Most studies have shown that these scoring systems are as efficient as the STONE score. Al Adl et al., in their prospective study, compared the four scoring systems and noted that they were equally efficient in predicting the stone-free rate [47]. Illahi Bux et al. compared the STONE, GSS, and S-ReSC models and concluded that they showed a high accuracy in predicting the outcome of single-tract PCNL [32]. Chen et al. also reported similar results [48]. In a study by Ayranci et al., the authors demonstrated a strong correlation between the CROES, STONE, and S-ReSC scoring systems in forecasting the completeness of stone clearance [49].

Several other studies have compared the STONE scoring system with other similar models and have shown them to be comparably predictive overall, with study-specific differences, in their predictive ability for stone-free rate and other surgical outcomes [50-53]. None of the scoring systems has shown any significant superiority over others in predicting stone-free rates; however, each has peculiar strengths and weaknesses [7,20]. An interesting advantage of the STONE scoring system is that the STONE score, as modified by Molina et al., takes into cognizance the different positions of stones within the ureter and therefore can be used in studies among patients with renal and ureteric stone disease undergoing URS [9].

In a prospective study conducted between January 2017 and June 2018 involving 102 patients, the STONE score was calculated using data from preoperative non-contrast CT scans, with stone-free status evaluated at four weeks postoperatively. The results demonstrated that the STONE score predicted stone-free rates with an impressive accuracy of 88%. A cut-off score of 8 was found to be particularly effective for predicting stone-free rates. Among the individual components of the score, stone size, pelvicalyceal obstruction, calyceal involvement, and stone density showed significant correlations with the stone-free rate, whereas tract length did not (p = 0.81) [54]. Although this study did not show any correlation with postoperative complication rates, its simplicity and predictive strength make the STONE score a practical tool in planning PCNL procedures and counselling patients regarding expected outcomes [54].

In a multi-institutional study, Al-Azab et al. evaluated the predictive power of a novel machine learning (ML) model in determining stone-free status after PCNL, comparing it against two conventional scoring systems: GSS and STONE [55]. The retrospective analysis included 320 PCNL cases, where data were fed into a supervised learning model using clinical and radiological parameters. The results showed that the ML model outperformed both GSS and STONE in predictive accuracy, achieving an AUC of 0.87 compared to 0.79 (STONE) and 0.75 (GSS). Notably, the ML system was more adaptable to patient variability and could integrate multi-dimensional data without the subjectivity inherent in traditional scoring. The authors concluded that ML offers a promising, data-driven supplement, or even alternative, to established nephrolithometric scoring tools, particularly in large clinical datasets or complex stone cases.

Labadie et al. conducted a retrospective study and comparison of three major nephrolithometric scoring systems, i.e., GSS, STONE nephrolithometry, and the CROES nomogram, in predicting outcomes of PCNL [56]. The retrospective study included 246 patients treated at a high-volume endourology center, with scoring performed independently by two blinded reviewers using preoperative CT imaging. The study found that all three systems had significant predictive value for stone-free rate, but the STONE score showed the highest correlation with both operative time and estimated blood loss. The Guy’s and STONE nephrolithometry scores were associated with estimated blood loss and length of stay predictions. The CROES nomogram did not predict estimated blood loss or length of hospital stay. The study concluded that the STONE scoring systems were adept in predicting stone-free states. However, the STONE score was noted for its objectivity and direct CT-based measurements, while the CROES nomogram incorporated a broader range of clinical parameters. The authors also concluded that while each scoring system has strengths, its clinical utility depends on the balance between predictive accuracy, reproducibility, and ease of use. The study provided key insights into tailoring the choice of nephrolithometric tool to institutional preferences and available imaging resources.

Furthermore, following a study by Singla et al., the comparison of the AUC of predicting the stone-free rate indicated that there was no remarkable difference between GSS, STONE nephrolithometry, and the CROES nomogram. Nonetheless, GSS was the only stone scoring system that predicted complications after PCNL [51]. Mushtaq et al. conducted one of the largest prospective studies evaluating nephrolithometric scoring systems specifically in the context of mini-PCNL [57]. The study included 410 adult patients who underwent mini-PCNL at a tertiary center in India. Three scoring systems, i.e., GSS, the STONE nephrolithometry score, and the S-ReSC score, were compared in terms of their ability to predict stone-free rates and complications. The study found that all three scores had significant predictive ability for the stone-free rate. Mean STONE scores were significantly higher in patients who had residual stones (7.92 ± 1.80) compared to those who achieved stone-free status (6.61 ± 1.20), highlighting its utility in operative planning. Although the predictive ability of these scores for complications was moderate (AUCs around 0.61-0.63), the study emphasized their value in preoperative risk stratification and patient counselling. This work contributes significantly to the validation of nephrolithometry systems in minimally invasive stone surgery.

In a prospective single-center study, Farhan et al. evaluated the clinical relevance of the STONE nephrolithometry score in predicting PCNL outcomes [58]. Conducted in Pakistan, the study involved 107 patients undergoing standard PCNL. The study aimed to validate the utility of this relatively new scoring system in a South Asian population. The findings demonstrated a statistically significant association between higher STONE scores and reduced stone-free rates, prolonged operative time, and increased hospital stay. Each component of the score, particularly stone size and number of calyces involved, was found to independently affect surgical outcomes. The study also observed that STONE scoring was easy to apply in routine practice, with strong interobserver agreement. Importantly, this study reinforced the role of STONE as a practical, objective, and reproducible scoring system that is especially useful in resource-limited environments where preoperative planning must be highly efficient.

Elkoushy et al. explored the global acceptance and perceived utility of various nephrolithometry scores through a structured international web-based survey involving 162 practicing endourologists [59]. This cross-sectional study aimed to assess the impact of scoring systems, such as the GSS, STONE, CROES, and S-ReSC, on clinical decision-making, particularly in terms of preoperative planning, predicting outcomes, and communicating risks to patients. The results revealed that while most clinicians were aware of these scoring systems, there was variability in usage patterns. GSS and STONE were the most frequently used tools, valued for their simplicity and clinical relevance. Interestingly, although the majority acknowledged the theoretical benefits of nephrolithometric scoring, a substantial number expressed concerns regarding interobserver variability and the time required for scoring, especially with complex systems such as CROES. The study highlighted the need for continued validation and standardization to improve global adoption. It also underscored the role of scoring systems not only in outcome prediction but also in fostering communication between clinicians and patients regarding surgical risks and expectations.

Kumar et al. conducted a prospective comparative study to assess the effectiveness of two commonly used scoring systems, STONE and GSS, in predicting surgical outcomes and complications following PCNL [60]. The study included 445 patients and assessed outcomes such as stone-free status, operative time, and complication rates. The findings showed that both scoring systems were effective in predicting stone-free rates, with higher scores correlating with lower success rates. The STONE score, however, demonstrated a slightly stronger association with operative time and intraoperative difficulty. While both scores were comparable in predicting postoperative complications, the authors favored STONE for its ease of application and objective criteria based on CT imaging. This study supported the practical utility of STONE in routine urological practice, especially in centers relying heavily on radiological parameters for operative planning. The authors concluded that both systems offer meaningful preoperative risk stratification and should be considered complementary tools in the PCNL workflow.

A systematic review published in Nature Reviews Urology further substantiated the equivalence of the four main nephrolithometry systems, concluding that while all predict stone-free rates with similar accuracy, each exhibits distinct strengths. The GSS, for instance, is better at forecasting complications; STONE offers a strong balance of simplicity and predictive accuracy; and the CROES nomogram is particularly informative in complex cases, such as chronic kidney disease or anatomical variants [7].

Akçay et al. retrospectively evaluated the predictive accuracy of three nephrolithometric scoring systems, i.e., GSS, STONE score, and the CROES nomogram, in 289 patients undergoing PCNL [61]. Their goal was to determine which system most reliably predicted the stone-free rate and surgical complexity. All three systems demonstrated significant predictive value, but the STONE score had the highest accuracy (AUC = 0.815), followed closely by CROES (0.793) and GSS (0.780). Higher scores were associated with lower stone-free rates and longer operative times. The authors concluded that while each system has merit, the STONE score is particularly practical due to its simplicity and strong radiological basis. This study reinforced the value of nephrolithometric tools in PCNL planning and suggested that the STONE score offers a good balance of accuracy and ease of use in routine clinical practice.

A prospective observational analytic study conducted at Soetomo General Hospital, Surabaya, sought to compare the effectiveness of the Modified GSS and STONE scores in predicting the stone-free rate following PCNL [62]. Patients scheduled for PCNL were evaluated preoperatively with CT stonography to determine their STONE and GSS scores. Postoperative outcomes were assessed using KUB radiography to determine residual stone burden and evaluate the stone-free rate.

The findings of the study demonstrated a stronger correlation between the Modified GSS and stone-free rate compared to the STONE score. Specifically, statistical analysis revealed that GSS had a strong positive correlation with stone-free rate, with a strength value of 0.609 and a statistically significant p-value of 0.05. This implies that higher GSS grades, which indicate more complex stone morphology, were associated with lower stone-free outcomes. Conversely, the STONE score also showed a positive correlation with stone-free rate (relationship strength value of 0.55), yet this did not reach statistical significance (p = 0.228).

The conclusion drawn by Sanjaya et al. (2021) was that the Modified GSS is a superior predictor of stone-free outcomes following PCNL when compared to the STONE score. The GSS, by incorporating factors such as the anatomical location and complexity of the stone, appears to provide a more comprehensive estimation of surgical difficulty and outcome. These findings were found to be clinically relevant, particularly in centers performing PCNL on a high volume of patients with varying stone complexities. The use of GSS in preoperative evaluation may aid surgeons in better risk stratification, surgical planning, and improving informed consent discussions with patients. While the STONE nephrolithometric score remains a valuable tool, especially for quantifying stone burden and anatomical considerations, it may be less predictive when applied alone [62].

The STONE nephrolithometry score holds its ground well when compared to other established scoring systems. It reliably predicts stone-free outcomes and perioperative parameters such as operative time and blood loss, is simple to use, and has broad clinical applicability, including in patients with ureteric stones. While it may not outperform others in complex anatomical or renal impairment scenarios, its efficiency and ease of implementation make it a preferred tool in routine urologic practice. The growing body of comparative literature reaffirms the need for clinicians to select scoring systems not based solely on raw predictive power but on patient context, ease of use, and institutional workflow, domains where the STONE score consistently excels.

Limitations/weaknesses of the STONE scoring system

There is doubt about how well the score can be used in wide populations, as it was validated with a relatively small population size. This argument, however, is weakened by the increasing amount of literature that has validated this system. Moreover, the predictive accuracy of the model will be improved by standardizing ways of measuring the different parameters of the system, such as the size of the stone. This will bring uniformity and help avoid bias [20].

Recommendations for future research

Integration of artificial intelligence into urology stone imaging, thereby creating an easy way of assessing the score and providing ready access to clinical recommendations. This will make the clinical application of STONE scoring systems easy for the clinician. Further, there is a need for validation of larger and more varied groups of patients. This will make results generalizable among different population groups. Finally, an extensive study is required to establish its role in predicting complications, as several studies have reported variable outcomes.

## Conclusions

With the rise in the incidence of urological stone disease and the widespread use of lithotripsy, the formulation and use of clinical models to predict stone clearance and other outcome measures is vital. The STONE scoring system is a simple and efficient tool that can be easily applied in clinical practice. It can be used in preoperative planning, risk assessment for patient counselling, and standardized reporting of outcomes. It can, therefore, be a relevant tool for the practicing urologist.
